# Tissues and Tumor Microenvironment (TME) in 3D: Models to Shed Light on Immunosuppression in Cancer

**DOI:** 10.3390/cells10040831

**Published:** 2021-04-07

**Authors:** Teresa Ho, Rasha Msallam

**Affiliations:** 1 p53Lab, Agency for Science, Technology and Research (A*STAR), 8A Biomedical Grove, Neuros/Immunos, #06-04/05, Singapore 138648, Singapore; 2 Laboratory for Translational and Molecular Imaging (LTMI), Cancer and Stem Cell Biology Programme, Duke-NUS Medical School, 8 College Road, Singapore 169857, Singapore; 3 Cancer Immunotherapy Imaging “CITI” Programme, Duke-NUS Medical School, 8 College Road, Singapore 169857, Singapore

**Keywords:** organoid, immunosuppression, tumor microenvironment, MDSC, humanized mouse models

## Abstract

Immunosuppression in cancer has emerged as a major hurdle to immunotherapy efforts. Immunosuppression can arise from oncogene-induced signaling within the tumor as well as from tumor-associated immune cells. Understanding various mechanisms by which the tumor can undermine and evade therapy is critical in improving current cancer immunotherapies. While mouse models have allowed for the characterization of key immune cell types and their role in tumor development, extrapolating these mechanisms to patients has been challenging. There is need for better models to unravel the effects of genetic alterations inherent in tumor cells and immune cells isolated from tumors on tumor growth and to investigate the feasibility of immunotherapy. Three-dimensional (3D) organoid model systems have developed rapidly over the past few years and allow for incorporation of components of the tumor microenvironment such as immune cells and the stroma. This bears great promise for derivation of patient-specific models in a dish for understanding and determining the impact on personalized immunotherapy. In this review, we will highlight the significance of current experimental models employed in the study of tumor immunosuppression and evaluate current tumor organoid-immune cell co-culture systems and their potential impact in shedding light on cancer immunosuppression.

## 1. Introduction

Advances in cancer immunotherapy (CIT) continue to revolutionize our cancer therapy arsenal. To date, the most significant clinical breakthroughs have come from application of immune checkpoint inhibitors (ICIs) [1] and T cell-based adoptive cell transfer (ACT) such as chimeric antigen receptor (CAR) T cells [2]. This has led to FDA approval of cytotoxic T-lymphocyte-associated protein 4 (CTLA-4) and programmed death-ligand 1/programmed death-1 (PD-L1/PD-1) blockers, immunomodulatory cytokines and CD-19 targeting CAR T cells. While effective tumor eradication and long-term survival has been reported in a fraction of cancer patients, sustainable benefits of these immunotherapy strategies have yet to materialize for the majority. The roadblocks we face are multifold; primarily, tumors are highly heterogeneous as are the corresponding tumor immune microenvironments (TIME). Secondly, the tumor and the patient’s immune system and TIME are also constantly evolving throughout tumor development and in response to chemotherapy. Both systems are pre-equipped with multiple compensatory and feedback loops that enable therapy evasion and development of resistance. Thirdly, we lack definitive clinical biomarkers to identify patients who will benefit from targeted and/or combinatorial therapy as well as detect resistance mechanisms. Much progress has been made in identification of TIME immune components; how these correlate with tumor molecular subtypes and their implications for approved immunotherapies have been elegantly discussed in various reviews [2,3,4,5,6,7,8]. Potential biomarkers have also been proposed to predict patient response to immunotherapies such as expression of ligands and clonal tumor neoantigens such as PD-L1, PD-1, CTLA-4, beta-2-microglobulin (β2M) and class-I MHC (HLA-I) in determination of efficacy of checkpoint blockers [9] and expression of CD58 as a predictor of response to CD19 CAR T cell therapy [10].

However, we still do not fully understand the impact of the tumor–TIME interplay and how this shifts longitudinally over time and space. Understanding such crosstalk is crucial as we continue to unravel the role of immunosuppressive subsets, and given the urgent need to identify the cellular and molecular drivers of immune escape or immunosuppression to combat drug resistance and/or low efficacy. The tissue-specific contexts in which these tumor–TIME interactions occur are also obscure and current therapeutic approaches do not consider influences of different anatomical sites, be it for primary tumor development or metastases [11]. In this review article, we will focus attention on the biggest challenge to successful, sustainable cancer immunotherapy posed by immunosuppressive mechanisms and how technical advances in three-dimensional (3D) models are paving the way for a more in-depth understanding of the interplay between tumor and immune cells over traditional use of in vivo models.

## 2. Mechanisms of Cancer Immunosuppression

**Figure 1:**
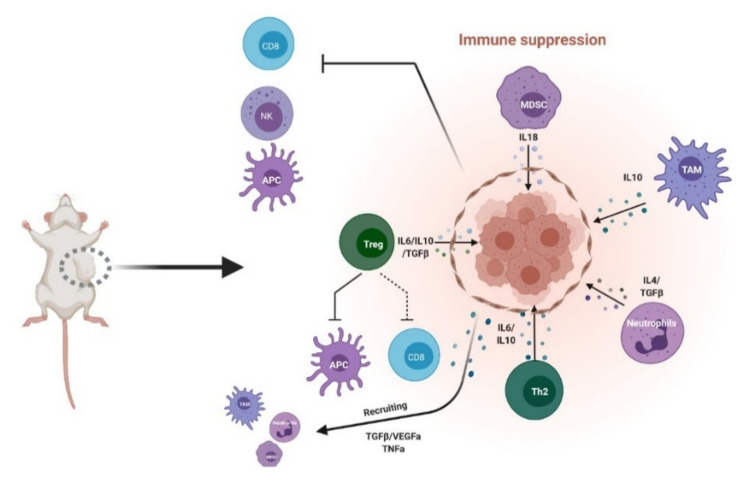
Inhibitory tumor immune microenvironment (iTIME). Schematic of known components of inhibitory TIME. MDSC: myeloid-derived suppressor cells; TAM: tumor-associated macrophages; Th2: helper T cells; Treg: regulatory T cells; NK: natural killer; APC: antigen-presenting cells; CD8: cytotoxic T cells. Created with BioRender.com (accessed on 6 April 2021).

The immunogenicity of a tumor is reflected by the capacity for an increase in tumor-infiltrating lymphocytes (TILs) and is often an indicator of therapy outcomes. However, recent studies have shown that the main challenges in realizing cancer therapy efficacy is not uniquely due to the low ratio of infiltrated TILs within the tumor; rather, the heterogeneity of the TIME, notably the inhibitory tumor immune microenvironment (iTIME), is a factor to be reckoned with [12]. Components of the TIME can influence tumor immunogenicity by either blocking recruitment of TILs and/or creating an inflammatory environment that transforms effector and cytotoxic T lymphocytes (CTLs) into a functionally “exhausted”, inactive (anergic) state. Under such circumstances, the tumor is classified as “cold” [13,14]. CITs such as anti-CTLA-4 (ipilimumab or tremelimumab) and anti-PD-1/PD-L1 (atezolizumab, avelumab, durvalumab, nivolumab or pembrolizumab) [15,16,17,18] have been touted to promote TIL infiltration alongside conventional chemotherapy and radiotherapy [5]. However, recent reports have highlighted that CIT efficacy cannot be defined by TIL infiltration alone and that the effects of other components of the iTIME need to be considered. Thus far, immunosuppressive cells such as neutrophils, regulatory T cells (Treg), monocytic/granulated-derived suppressor cells (MDSC and Gr-DSC, respectively), some subsets of tumor-associated macrophages (TAM) [19] and cancer-associated fibroblasts (CAF) [5,6,20,21] have been identified. The infiltration of such immunosuppressive subtypes has been shown to result in inflammatory signaling by tumor cells (TNFα, TGFβ, VEGFa, CXCL12) which, in turn, promotes further recruitment of immunosuppressive cells to the TIME in a cascading “wave”. These immunosuppressive subtypes are also capable of secreting soluble factors such as proinflammatory cytokines and chemoattractant such as IL10, IL35, IL6, IL13 and IL18, which reinforces an iTIME phenotype [22,23,24,25,26,27,28,29]. The combined effect is sufficient to impede CTL function and CIT efficacy, facilitating tumor development and/or metastatic progression. TILs have also been found to be inactivated or become functionally “exhausted” with reduced capacity in killing and clearance of tumor cells. Tumor expression of chemoattractant such as CXCR1 and CXCR2 results in recruitment and expansion of neutrophils and MDSCs within the TIME. Neutrophils are capable of forming neutrophil extracellular traps (NETs) that surround and shield the tumor from CTLs [30]. Similarly, the presence of Tregs surrounding the tumor exerts an inhibitory effect on antigen-presenting cells (APC) and effector T cells. The mechanisms by which Tregs inhibit APCs and effector T cells include direct interaction with these cells through TCR-MHCII, costimulatory signals such as CD80 or CD86/CTLA-4 or secreting inhibitory cytokines such as IL35 [31,32]. MDSCs and TAMs have more recently been implicated as significant contributors to an iTIME. However, these cell types are extremely heterogeneous and further in-depth studies are required to verify their importance as well as identify the mechanisms by which they contribute to immunosuppression. Characterizing and understanding the multifaceted nature of the iTIME is still in its infancy. While efforts have led to the identification of multiple immunosuppressive cell types, we need further development and improvement of current in vivo models to not only realize the whole spectrum of cell types and immune–tumor crosstalk events that contribute to the iTIME landscape, but also to dissect the role of the different players (Figure 1).

## 3. Tumor-Driven Mechanisms of Cancer Immunosuppression

**Figure 2:**
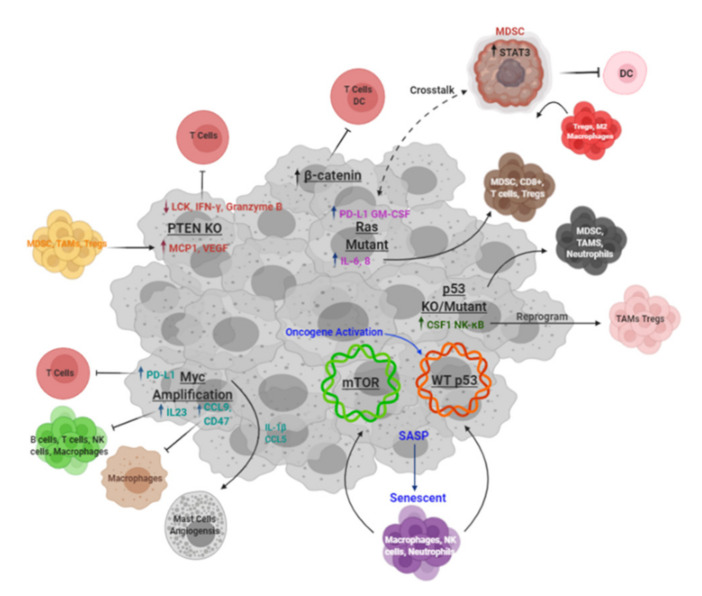
Tumor-driven mechanisms of immunosuppression. Schematic of known mechanisms of immunosuppression driven by key oncogenes and tumor suppressors. Created with BioRender.com (accessed on 6 April 2021).

Large-scale sequencing analyses of patient tumors across multiple tissue types have revealed potential correlations between mutational load and aberrant expression of driver oncogenes and tumor suppressors and the immune subtypes or infiltrate present in the TIME. Here, we briefly highlight major signaling pathways perturbed in tumor development and their direct and indirect effects on promotion of an immunosuppressive TIME (Figure 2).

### 3.1. TP53

Loss or mutation of TP53 is a major driver of tumor development and its effect on the TIME has been studied extensively and expertly reviewed elsewhere [4,33,34,35,36]. We highlight below some key findings pertaining to cultivation of an immunosuppressive TIME as well as give mention to the significant role of p53 in oncogene-induced senescence and its unique effects on the TIME. The clearest indications of an immunosuppressive TIME in the absence of mutation of p53 have been demonstrated in cancer models of the breast, ovary, prostate, pancreas, lung, skin and blood. P53 loss has been strongly associated with recruitment of pro-tumor myeloid cells [37], tumor-associated macrophages (TAMs) [33] and even an overall increase in circulating neutrophils that support tumor metastasis [38]. Several reported mouse models of breast cancer and corresponding analysis of human datasets showed that mutant p53 tumors were characterized by increased macrophage-specific colony-stimulating factor (CSF1) and signaling [39]. P53 mutant tumor cells also influence the reprogramming of recruited macrophages and myeloid cells through secretion of cytokines and chemokines [40]. In certain tumor types (namely prostate, ovarian and pancreatic), p53 loss has been shown to modulate differentiation of regulatory T cell (Treg) populations which suppress effector T cells [7,41]. In response to oncogene activation, p53 activation results in cell cycle arrest and induction of senescence. This is accompanied by induction of a senescence-associated secretory phenotype (SASP) where macrophages, natural killer (NK) cells and neutrophils are recruited to assist in removal of senescent cells [42]. In an H-RAS model of liver carcinoma, reactivation of p53 in tumor cells upregulated chemokines such as CCL2, CXCL1 and CXCL2, which promoted NK cell recruitment and tumor clearing [43].

Mammalian target of rapamycin (mTOR) signaling can also induce SASP with inhibition by rapamycin attenuating the influx of macrophages, T, B and NK cells into N-RAS mutant liver tumors [44,45]. These findings indicate that while inhibition of mTOR may affect tumor growth, it may also reduce inflammation and clearance of senescent cells. In-depth investigations are required to determine the impact of targeted therapies like rapamycin on TIME, and whether there might be tissue- or stage-context-dependencies. Furthermore, we need to better determine whether therapeutic modulation of SASP might result in chronic inflammatory phenotypes that can inadvertently promote tumor progression [46].

### 3.2. NF-κB

NF-κB is a transcription factor that regulates cell proliferation and survival; classical targets include tumor necrosis factor (TNF), IL-1β, IL-6 and other proinflammatory mediators [47]. Crosstalk and co-regulation between NF-κB and p53 have been reported [48], with potential implications for remodeling of the TIME. Mutant p53 has been shown to upregulate NF-κB signaling in a “gain-of-function” (GOF) manner, influencing macrophage function. The hotspot R248W p53 mutant, which is found in multiple tissue types, induces NF-κB-dependent exosome secretion of miR-1246, which in turn reprograms macrophages to promote tumor growth [49]. Azoxymethane (AOM)-induced colorectal tumorigenesis was enhanced in a p53-deficient background partly due to an NF-κB-driven inflammatory TIME [50]. Genetic ablation of IKKβ, a protein involved in NF-κB activation, in cancer cells or immune cells significantly reduced tumor proliferation and invasion by impairing cytokine production. This is supported by similar observations of tumor growth impairment and increased anti-tumor immune cell infiltration following NF-κB inactivation in a KRAS/TP53 model of lung carcinoma [51].

### 3.3. Wnt Signaling

Wnt-β-catenin signaling is a well-known key driver of tumor development and has been implicated in TIME modulation. In a Braf/Pten/β-catenin mouse model of melanoma, β-catenin-positive tumors demonstrated a significant decrease in T cell infiltration compared to β-catenin-negative counterparts [52]. These in vivo findings correlated with those in human metastatic melanoma. Analysis of The Cancer Genome Atlas (TCGA) patient samples revealed similar trends between dysregulated β-catenin signaling and exclusion of T cells from the tumor microenvironment (TME) across multiple solid tumor types [53]. In terms of elucidating probable mechanisms, active β-catenin has been shown to reduce chemokine production, affecting the recruitment of CD103+ cross-presenting dendritic cells (DC) which are critical in priming anti-tumor T cell responses [54].

### 3.4. PTEN

The tumor suppressor PTEN is largely responsible for modulating Akt activation via phosphatidylinositol 3-kinase (PI3K) activity. In general, PTEN loss has been associated with reduced infiltration of CD8+ T cells and poorer prognosis. Resultant tumors also present with reduced expression of LCK, a T cell-specific protein and effector molecules like IFN-γ and granzyme B [55]. This is supported in preclinical melanoma models where treatment of PTEN-deficient tumors with an agonist against a T cell costimulatory molecule, OX40 and the PI3K inhibitor, GSK2636771 [56], enhanced the anti-tumor immune response by promoting CD8+ T cell infiltration [57]. A melanoma mouse transplant model also put forward an alternative mechanism of immunosuppression where PTEN-deficient tumors promoted recruitment of immunosuppressive cells such as macrophages, regulatory T cells (Treg) and myeloid-derived suppressor cells (MDSC) through upregulation of monocyte chemoattractant protein-1 (MCP1/CCL2) and vascular endothelial growth factor (VEGF) [58].

### 3.5. MYC

MYC is a master regulator of cell proliferation and differentiation and a frequently amplified oncogene in a variety of cancers. In a mutant KRAS lung adenocarcinoma model, conditional MYC amplification led to increased expression of IL-23 by tumor cells that inhibited recruitment of intra-tumoral B, T and natural killer (NK) cells [59]. The increased expression of CCL9 instead recruited macrophages that promoted angiogenesis and inhibited T cell functions. These tumors were found to be dependent on MYC amplification and subsequent inactivation of MYC saw tumor regression in an NK cell-dependent manner. Meanwhile, in an MYC lymphoma mouse model and in vitro cell lines, MYC was shown to transcriptionally upregulate PD-L1 and CD47 in tumor cells [60,61]. PD-L1 interaction with PD-1 on CD4+ T cells attenuated signaling where CD47 binding to macrophages inhibited their phagocytic ability. Interestingly, in a model of neuroblastoma, N-MYC amplification resulted in downregulation of PD-L1. This contrast was attributed to MYC suppression of proinflammatory signaling and interferon production [62]. Further understanding of the tissue-specific differences of similar perturbations in genetic and signaling pathways and how these influence the immunosuppressive profile of the TIME will be critical in furthering therapeutic efforts. The function of CD4+ T cells was demonstrated in an MYC T cell acute lymphoblastic lymphoma (T-ALL) model where MYC inactivation correlated with an increase in expression of the cytokine Thrombospondin-1 (TSP-1), leading to induction of senescence and inhibition of angiogenesis by intra-tumoral CD4+ T cells [63]. In a pancreatic β-cell cancer mouse model with inducible expression of a dominant-negative MYC mutant, the study found that inhibition of MYC effectively reduced the degree of infiltrating macrophages and neutrophils, directly impacting tumor regression [59]. Conversely, MYC expression in β-cells promoted production of proinflammatory cytokines such as CCL5 and interleukin-1β (IL-1β), which facilitated angiogenesis and recruitment of pro-tumoral mast cells [64,65].

### 3.6. RAS

Mutations in the RAS family genes are common drivers in multiple tumor types. Mutant RAS is capable of regulating expression of cytokines such as IL-6 and IL-8 in a variety of in vitro and in vivo models leading to tumor progression and infiltration of multiple immune cell types such as myeloid cells, CD8+ T cells, Tregs, IL-17-producing lymphocytes (as reviewed elsewhere [66,67,68]). Such changes in cytokine profiles have been reported to result in accumulation of CD11b+Gr1+ immunosuppressive cells in a variety of tumor models, of which lung and pancreatic have been most studied [69]. Interactions between mutant RAS and p53 signaling pathways have also been shown to have an “additive” effect on expression of PD-L1 [70,71] and cytokines such as granulocyte–macrophage colony-stimulating factor (GM-CSF) [72] and their resultant impact on recruitment of immunosuppressive cells.

Another significant angle of recent investigations pertains to the effect of RAS signaling in tumor cells on activation of key pathways such as STAT3 and NF-κB in surrounding intra-tumoral immune cells. These pathways are also known downstream nodes of RAS signaling. Activation of STAT3 in MDSCs led to induction of Tregs, DC inhibition and macrophage polarization towards the pro-tumorigenic M2 subtype in a KRAS lung cancer model [73]. Conversely, depletion or attenuation of STAT3 signaling in myeloid populations promoted anti-tumor immune responses in the form of CD8+ T cells and suppressed tumor development. Likewise, activation of NF-κB in surrounding myeloid populations and macrophages influenced the inflammatory cytokine repertoire of the TIME, promoting lung cancer progression [74]. Moving forward, more efforts to study this crosstalk between epithelial and immune cell signaling mechanisms are critical in advancing our understanding of tumor development and design of combinational therapy.

### 3.7. Moving Forward

Collectively, the findings reviewed above demonstrate the unique effect of cell-intrinsic genetic modifications on the composition of the surrounding TIME as well as the spectrum of pro- and anti-inflammatory factors produced within the tumor and TIME. Currently, the majority of these studies have largely focused on the effect of perturbing a single genetic pathway in a given mouse tumor model. However, tumors are not only genetically heterogeneous but are essentially also a collection of multiple mutant clonal populations. Depending on the predominant signaling and molecular pathways governing each of these clonal populations, the crosstalk and interactions between these clonal populations will also affect the fundamental nature of the TIME. More recently, studies comparing independent mouse models of lung [75] and prostate [41] cancer revealed remarkable, yet perhaps unsurprising, differences in the immune cell repertoire of the TIME. While these findings highlighted the importance of in vivo mouse models in identification of the components of the complex TIME, we still need mechanistic insight into the functionally significant, reciprocal interactions between tumor and immune cells and how they evolve with tumor progression and therapeutic and clinical interventions. Only then would we be able to exploit the genetic aberrations and TIME of a tumor for design of more personalized interventions.

## 4. Overview of Current Experimental Models in Cancer

### 4.1. Mouse Models

#### 4.1.1. Syngeneic Mouse Models

Genetically engineered mouse models (GEMMs) have long been a staple in cancer biology. GEMMs where one or more cancer-driving genes have been modulated (knock out or mutant), leading to spontaneous tumor development, have allowed us to gain insight into tumorigenesis and tumor development [76]. Similar genetic manipulation of immune-related genes (i.e., CD5KO, IFNγKO) has allowed us to better understand the TIME and mechanistic tumor-immune interactions that contribute to tumor development [77,78]. Mouse models are pertinent in studying immunosuppression in cancer, and can be broadly categorized by their immunity status.

Syngeneic or allograft mouse models have essentially fully functional immune systems (immunocompetent), and involve introduction of murine tumor tissue subcutaneously (s.c.) or orthotopically (i.e., same primary anatomical location) from a similar genetic background. For instance, melanoma is often studied in such mouse models by injecting B16F10 subcutaneously (s.c.) in localized skin melanoma models or intravenously (i.v.) in metastatic models [79]. By using this for in vivo systems, it was found that melanoma exhibited a suppressive TIME phenotype that limited CD8+ T cells activation and promoted the recruitment of MDSC via CCR2 and GM-CSF-dependent mechanisms [80]. Another study employing syngeneic models had reported the variability between MDSC subsets (granulocytic (G-MDSC) or monocytic (MDSC)) and their enrichment within the TIME and demonstrated that this was dependent on not just the type of cell line, but also the genetic background of the recipient mouse [81]. In this study, it was shown that thymoma (EL4 cells), melanoma (B16F10 cells) engrafted in C57BL/6 and colon cancer (CT26 cells) in BALB/c mice exhibited high accumulation of G-MDSC and less MDSC in an immunosuppressive TIME. Such studies highlight the particularity of the chosen mouse model and cell lines employed, adding further complexity to investigating and resolving the role of MDSC by using such mouse models (Table 1).

However, syngeneic mouse models are largely still considered as not fully representative of tumor development in humans, given the distinctions between human and murine immune systems. Furthermore, syngeneic models do not fully recapitulate the in situ TIME landscape of human tumors. Recent studies have pointed out the intrinsic variation between GEMMs where the most studied oncogenes (KRAS which is responsible for 30% of all human cancers [82] and c-ErbB-2 or HER-2 which is amplified in 30% of all breast cancers and is predominant in head and neck cancers [83]) and tumor suppressors (TP53 which is lost or mutated in more than 50% of all cancers [84]) have been modulated and the human condition. Fundamental distinctions in the molecular signaling mechanisms, mutational processes and rates and even the timescale of tumor development all have a significant impact not only on the course of tumor development but the interactions between the tumor and TIME.

#### 4.1.2. Humanized Mouse Models

The development of humanized mouse models (SCID and NOG (NOD/Shi-scid/IL-2Rγnull)) has allowed the introduction of human tumor tissue [90,91]. As these mice lack T cells and B cells, rejection of transplanted material is prevented. Immunodeficient mice (hu-NOG) can be generated through engrafting NOG mice with human fetal liver or human stem cells (HSC or hCD34+) isolated from cord blood or bone marrow [92]. These mice develop a naïve human immune system, allowing the study of the TIME in vivo after transplanting commercialized human cancer cell lines or patient-derived xenografts (PDX).

Although humanized mouse models are relatively similar to syngeneic models due to presence of a reconstituted (hence semi-competent) immune system, they have been considered valid for bridging the gap between syngeneic mouse models and patients. In the case of PDXs, the serial passaging of patient-derived tumor cells in mice have been suggested to promote creation of a TME that is more reflective of that in patients [93]. As a result, PDXs have been accepted as an efficient tool in precision medicine to evaluate the efficacy of selected treatments. In breast cancer, PDX models have offered interesting insights into the increase in TGFβ secretion within the TIME of triple-negative breast cancer (TNBC) [94], as well as the increased recruitment of heterogeneous MDSC populations within the intra-tumoral TIME [95]. Furthermore, other PDX models have demonstrated the mechanisms behind how tumor cells can hijack the immune response by modulating the polarization of macrophages within the intra-tumoral TIME, inhibiting and polarizing them toward the immunosuppressive TAM phenotype [96].

However, humanized mice and PDXs are costly. While immunodeficiency is necessary in PDXs to prevent eradication of the implanted tissue material, it prevents further study of inflammatory processes which is critical in immunosuppression. Furthermore, in hu-NOGn models, the reconstitution of human stem cells is slow (up to 12 weeks) with high potential of developing graft versus host diseases (GvHD). PDX models can also introduce complexity and bias due to the need to passage tumors for several generations. The resultant loss of around 30% of human tumor cells on average and the subsequent impact on the TME are also confounding factors [97]. Furthermore, the introduction of different human tumor types into murine models often involves subcutaneous (s.c.) injection of tumor cells or patient-derived cell lines. This is preferred due to easy access and observation of tumor growth and alignment with ethical and animal welfare guidelines. Subsequent inferring of interactions between these s.c. tumors and immune components, while valid in relation to subcutaneous locations, is not representative of the actual tumor-immune interactions that characterize the tumor type in question. This is because of the inherent importance of the anatomical site on tumor behavior (orthotopic) and the TME landscape. Discrepancies between preclinical and clinical studies in translational applications also highlight such challenges. It has been shown with most cancers that drug candidates arising from murine xenograft models have poor clinical translation potential [98]. The efficacy of targets identified for CIT such as anti-PD-1/anti-PD-L1 has been found to be dependent on occurrence of tumor metastasis in other organs such as the liver [99]. This undoubtedly raises another question about the importance of remodeling the cancer environment in its “organ of origin” (orthotopical) instead of s.c. for a more realistic investigation of tumor–TIME dynamics. Such variability could be further compounded by genetic differences between human and mouse as well as the practical methods employed in murine models (i.e., dose, the route of administration and the frequency of applied treatment protocols).

**Figure 3:**
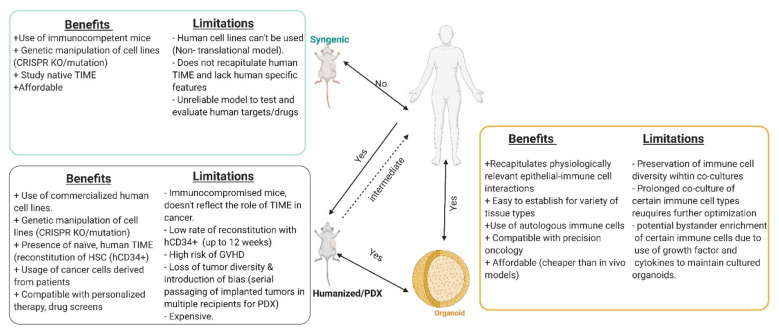
Comparison of the benefits and limitations of in vitro and in vivo experimental models. Created with BioRender.com (accessed on 6 April 2021).

Ultimately, in vivo murine models have contributed substantially to our fundamental understanding of tumor development and microenvironmental considerations (Table 1). However, the challenges faced when trying to clinically translate findings has emphasized the fundamental differences between human and murine TIMEs. Differences in representative immune cell types, nature of tumor evolution, tumor–cell interactions and even gradients in drug penetration in tumors (dependent on use of transplantation or orthotopic models) all significantly impact translatability. Tumor cells exhibit high plasticity and mutual interactions between the tumor and its immune and stromal microenvironment result in a formidable “ecosystem” shaped over time that is tolerant to therapy [97,100]. Better models that track such developments over time, recapitulate physiological processes and are feasible for personalization and scale are much needed (Figure 3).

While aforementioned mouse models have given us valuable insight into the dynamics between tumor and immune players within the TME and their impact on tumor development and therapeutic challenges, significant gaps still exist, in part due to inherent genetic differences between human patients and murine models as well as the inability to model the full spectrum of the human TME in mice.

### 4.2. 3D Models and Co-Cultures—Engineering Complexity to Mirror Physiology

The acknowledgment of the importance of tissue structural context, fueled by technological development, has led to establishment of in vitro 3D models in cancer biology. These 3D models exist either in the form of “spheroids” or mini-organs, also known as organoids. Constant advancements in tissue culture technology and our understanding of the TME have led to the ability to co-culture epithelial, immune and stromal cells, further mirroring the complexity of tissue systems in vivo. Below, we outline key technological advances in 3D models and their potential in focused, mechanistic study of the crosstalk between epithelial and immune cells as it pertains to immunosuppression.

#### 4.2.1. Spheroids

Spheroids are essentially self-assembling aggregates of tumor cells in the absence of a scaffold. These 3D, multicellular structures are a step up in complexity over 2D cell culture systems. With cells growing in direct contact, spheroids model the intercellular signaling as well as different proliferation rates and access to nutrients that one expects in a tumor mass [101].

**Figure 4:**
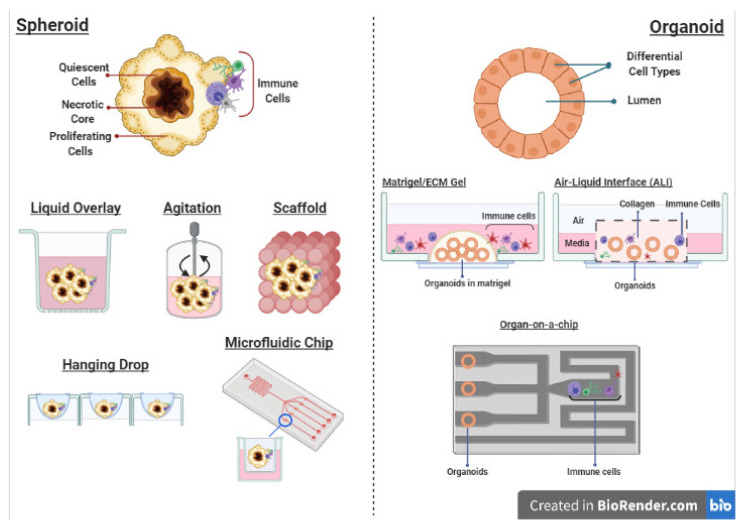
Different culture methods for 3D spheroids and organoids that are compatible with the co-culture of immune cells. Created with BioRender.com (accessed on 6 April 2021).

Various methods for culture of spheroids primarily involve application of low-adhesion surfaces or gravity to promote cell–cell interactions. These include liquid overlay, agitation-based cultures, the “hanging drop” method, cell seeding within scaffolds and microfluidic devices (Figure 4).

The simplest method—the liquid overlay system—involves coating non-adherent culture surfaces with agarose, poly(dimethylsiloxane) (PDMS) or poly-2-hydroxyethyl methacrylate (poly-HEMA) to prevent cell attachment [102]. Agitation-based cultures involve maintenance of cells in suspension in spinner flasks or rotary culture systems, continuously agitated to promote cell–cell interactions and present attachment to surfaces [103]. The “hanging drop” method is relatively rapid and employs the physical force of gravity to drive aggregation of cells at the tip of suspension droplets [104]. The development of patterned surfaces using micromolding or photolithography has allowed for generation of spheroids of defined sizes [105]. Furthermore, when used in conjunction with microfluidic devices, the generation of arrays of microwells facilitates use of spheroids in high-throughput phenotypic screening for therapies.

The co-culture of spheroids and immune cells has largely been employed with respect to drug screening, with most studies using “homogenous” cancer cell lines [106]. Recent studies have focused on patient-derived spheroids with introduction of multiple immune cell types. The co-culture of colorectal tumor spheroids with NK and T cells demonstrated the ability of immune cells to infiltrate with physiological effects on overall spheroid viability [107]. In response to immune infiltration, tumor cells upregulated HLA-E, a ligand of NKG2A, the inhibitory receptor expressed by NK and CD8 T cells. In another model involving spheroids derived from non-small cell lung cancer (NSCLC) in co-culture with the monocytic line THP-1 and peripheral blood monocytes (PBMCs) and cancer-associated fibroblasts (CAFs), spheroid infiltration of said monocytes and their subsequent polarization into an M2 macrophage phenotype (CD68+, CD163+ and CD206+) was observed [108]. This was accompanied by physiologically reproducible creation of an immunosuppressive TIME with secretion of cytokines such as IL4, IL10, IL13, CCL22, CCL24 and CXCL1. These studies demonstrate that spheroid-immune co-cultures are capable of capturing the physiologically relevant crosstalk between tumor and immune cells. However, while spheroids capture the 3D nature of tumors in vivo, the representation of physiological epithelial cell types is absent—a feature that renders 3D organoids a superior approach.

#### 4.2.2. Organoids

Organoids or “mini organs” recapitulate multiple structural aspects of a physiological tissue. The maintenance and differentiation of diverse cell types, preservation of cell polarity and cell–cell junctions and interactions and retention of genetic and epigenetic diversity of the tissue it was derived from all render organoids a powerful tool to model and study the complex interactions between tumor and immune cells [109]. Furthermore, the ease with which they can be generated from murine and human tissues alike as well as from induced pluripotent stem cells (iPSCs) facilitate establishment of such cultures from the outset [110].

With constant advancements in organoid technologies, culture and media formulations and scaffolding or microfluidic methodologies, the ease, accessibility and translatability of organoid systems have greatly improved. Their use as patient avatars in personalized therapy drug screens [111] has demonstrated that organoids are able to model and predict patient responses. In particular, the derivation of organoids from patient tumors (patient-derived organoids or PDOs) or iPSCs in colorectal [112,113], breast [114,115,116], liver [117,118,119,120], pancreatic [121,122,123,124] and neurological cancers [125,126,127,128,129,130] have collectively demonstrated the scalability of organoids in vitro, their ability to recapitulate tumor phenotypes and maintain genetic backgrounds and offer significant prognostic value of various therapeutic interventions. The further incorporation of immune cells in co-culture with PDOs would greatly improve physiological significance and prognostic impact of functional drug screens, especially in the context of immune checkpoint blockades [131].

The most common form of organoid culture involves culturing them in a “dome” of 3D Matrigel, submerged in tissue culture medium supplemented with additives (Wnt3a, R-spondin, epidermal growth factor (EGF) and Noggin) depending on tissue type (Figure 4). This sustains stem cell renewal and differentiation which allows for long-term culture of organoids [132]. Such a system of culture primarily enriches epithelial (normal or tumor) cells but does not often sustain stromal or immune components, which have to be added exogenously. However, studies have demonstrated that once introduced, autologous stromal and immune cells are capable of influencing organoid growth and other characteristics in a physiologically relevant manner [133]. This has been best exemplified in the context of intestinal cancers. Gastric organoids derived from murine and human tissue and co-cultured with CD8+ CTLs and DCs have been employed to elucidate important mechanistic insights into PD-L1 regulation in gastric tumor progression [134,135,136]. Such a system also allowed for convenient introduction of microbes such as *Helicobacter pylori* (*H. pylori*), a significant risk factor for gastric cancer, and study of corresponding effects on PD-L1 expression [137]. Similar co-cultures using patient-derived pancreatic [138], colorectal and non-small cell lung cancer organoids and tumor-reactive T cells allowed for detailed investigation of how the immune microenvironment drives tumor development and therapy resistance [139,140].

In an alternative method known as air–liquid interface (ALI) culture, organoids are embedded in a collagen or extracellular matrix-based gel in a transwell plate. Medium in an outer plate diffuses through the permeable transwell to the inner dish. The top of the gel layer is exposed to air that allows access to sufficient oxygen. Such a system allows for culture of tissue (normal or tumor)-derived organoids together with native TME components without the need to reconstitute cultures [141,142] and is capable of maintaining diverse native stromal and immune cell populations for several months [131]. This is an advancement in dealing with caveats of co-cultures involving accuracy when immune cell types are being introduced and avoiding biases in immune cell compositions. A recent study demonstrated the ability of the ALI system to capture cellular diversity as well as the physiologically relevant phenotypes of individual cell types [131]. The authors successfully co-cultured patient-derived tumor organoids with native immune cells (cytotoxic T cells, B cells, NK cells and macrophages) and observed T cell infiltration with corresponding expression of PD-1 and exhausted T cells. The profiles in culture mirrored those in fresh tumors. In similarly “holistic” 3D microfluidic cultures, organoids recapitulated therapeutic sensitivity to immunotherapy as in vivo human and mouse tumors [143].

Going beyond co-cultures, sophisticated methods such as ALI, microfluidics systems or “organs-on-a-chip” provide an environment where different cell populations can be cultured in defined regions that are interconnected by fluid channels that allow constant circulation of cells [144]. This allows for more in-depth investigation of the complex interactions between tumor and immune cells. Novel 3D bioprinting methods have facilitated the generation of dynamic scaffold matrices that mirror those in physiological tissues [145]. In a study employing glioblastoma organoids in a microfluidics chamber with macrophages, the polarization of macrophages towards an immunosuppressive M2 phenotype through TGFβ secretion and promotion of angiogenesis within endothelial-lined vascular channels was observable [146]. The ability to track cells live in such systems using fluorescent markers is also an added benefit [147]. The success of such co-culture systems in studying inflammation in cancer has also been reviewed elsewhere [148]. There has also been recent compelling evidence for epigenetic regulation of tumor immunosuppressive effects. The use of EZH2 and DNMT1 methyltransferase inhibitors led to increased CXCL9 and CXCL10 expression by ovarian tumor cells which promoted recruitment of CD8+ T cells. This exhibited a synergistic effect with PD-L1 blockade therapy and adoptive T cell transfusion in vivo [149]. Similar findings were reported in a melanoma model [150].

## 5. Future Perspectives

Cancer therapies have advanced substantially over the past decade and the increase in targeted therapies and immunotherapies in preclinical and clinical trials hold much promise. Understanding and comodulating the intra-tumoral TIME is also emerging as a hallmark of efficient cancer therapy. Immunosuppression poses a serious barrier to drug penetration and efficacy as well as infiltration of relevant anti-tumor effector cells. The constant evolution of the iTIME and the tumor, the heterogeneity of immune cell subtypes and the contributory role of mutations and/or signaling perturbations at the level of the non-immune TME (i.e., stromal component) are all critical factors that require urgent investigation. Thus far, in vivo mouse models have allowed us to garner much insight into the complexity of the TIME in tumor development but there has been sufficient evidence for their inherent limitations in fully reflecting the nature of human tumorigenesis and tumor development. With the advent of personalized targeted and combinatorial immunotherapy, there is great need for more efficient and cheaper patient avatars amenable to rapid, high-throughput screening. As in vitro 3D culture systems and technologies advance, the ability to capture and maintain the physiological accuracy and diversity of the patient tumor and TIME is increasingly improving. These 3D systems are also highly amenable to large-scale multi-omics studies and real-time imaging analyses. Not only will this facilitate validation of in vivo findings from mouse models, but it will also allow for fresh in-depth analysis of the unique roles of different TIME components and their interactions with the epithelium as well as drug screens.

Cancer is a past, present and future human and societal burden. We now have the tools to begin to advance our understanding of tumor immunology and how it shapes tumor development in order to develop novel biomarkers and clinical models to guide our approaches to diagnosis, prognosis and therapy.

## Figures and Tables

**Table 1:** Key tumor-associated immune cell types that contribute to an immunosuppressive TIME.

Tumor Model	Immune Cell Types Associated with Poor Survival	References
Lung	tumor-associated macrophages (TAMs), T-regulatory cells (T-regs), myeloid-derived suppressor cells (MDSCs)	[85,86,87]
Melanoma	tumor-associated macrophages (TAMs), T-regulatory cells (T-regs), myeloid-derived suppressor cells (MDSCs)	[88]
Gastric	T-regulatory cells (T-regs), myeloid-derived suppressor cells (MDSCs)	[89]

## Data Availability

Not applicable.
